# Resting-state networks and anosognosia in Alzheimer’s disease

**DOI:** 10.3389/fnagi.2024.1415994

**Published:** 2024-06-05

**Authors:** Manuela Tondelli, Daniela Ballotta, Riccardo Maramotti, Chiara Carbone, Chiara Gallingani, Clare MacKay, Giuseppe Pagnoni, Annalisa Chiari, Giovanna Zamboni

**Affiliations:** ^1^Department of Biomedical, Metabolic and Neural Sciences, University of Modena and Reggio Emilia, Modena, Italy; ^2^Neurologia, Azienda Ospedaliero Universitaria di Modena, Modena, Italy; ^3^Oxford Project to Investigate Memory and Ageing (OPTIMA), Experimental Medicine Division of Radcliffe Department of Clinical Medicine, University of Oxford, Oxford, United Kingdom; ^4^Department of Physics, Informatics and Mathematics, University of Modena and Reggio Emilia, Modena, Italy; ^5^Department of Mathematics and Computer Science, University of Ferrara, Ferrara, Italy; ^6^Wellcome Centre for Integrative Neuroimaging, Department of Psychiatry, University of Oxford, Oxford, United Kingdom

**Keywords:** anosognosia, unawareness, Alzheimer, dementia, mild cognitive impairment, salience, default-mode, resting state networks

## Abstract

**Background:**

Recent evidence suggests that anosognosia or unawareness of cognitive impairment in Alzheimer’s Disease (AD) may be explained by a disconnection between brain regions involved in accessing and monitoring information regarding self and others. It has been demonstrated that AD patients with anosognosia have reduced connectivity within the default mode network (DMN) and that anosognosia in people with prodromal AD is positively associated with bilateral anterior cingulate cortex (ACC), suggesting a possible role of this region in mechanisms of awareness in the early phase of disease. We hypothesized that anosognosia in AD is associated with an imbalance between the activity of large-scale resting-state functional magnetic resonance imaging (fMRI) networks, in particular the DMN, the salience network (SN), and the frontoparietal network (FPN).

**Methods:**

Sixty patients with MCI and AD dementia underwent fMRI and neuropsychological assessment including the Anosognosia Questionnaire Dementia (AQ-D), a measure of anosognosia based on a discrepancy score between patient’s and carer’s judgments. After having applied Independent Component Analysis (ICA) to resting fMRI data we performed: (i) correlations between the AQ-D score and functional connectivity in the DMN, SN, and FPN, and (ii) comparisons between aware and unaware patients of the DMN, SN, and FPN functional connectivity.

**Results:**

We found that anosognosia was associated with (i) weak functional connectivity within the DMN, in posterior and middle cingulate cortex particularly, (ii) strong functional connectivity within the SN in ACC, and between the SN and basal ganglia, and (iii) a heterogenous effect concerning the functional connectivity of the FPN, with a weak connectivity between the FPN and PCC, and a strong connectivity between the FPN and ACC. The observed effects were controlled for differences in severity of cognitive impairment and age.

**Conclusion:**

Anosognosia in the AD continuum is associated with a dysregulation of the functional connectivity of three large-scale networks, namely the DMN, SN, and FPN.

## 1 Introduction

Patients with Alzheimer’s Disease (AD) may be unaware of their cognitive and behavioral symptoms even in the early phases of the AD continuum, including the phase indicated as Mild Cognitive Impairment (MCI). The inability to recognize or adequately appreciate the severity of one’s own cognitive or behavioral disturbances is termed “anosognosia” or “impaired self-awareness” (Prigatano, 2009). Studies have reported that about 10% of patients with mild dementia have anosognosia and that this proportion increases up to 80% in patients with severe dementia (Orfei et al., 2010; Hanseeuw et al., 2020), suggesting an association of anosognosia with dementia severity. Anosognosia in patients with MCI and AD has significant clinical implications for their care, reduces compliance to treatment, threatens patient’s safety, and increases caregiver burden (Starkstein et al., 2007). Therefore, a better knowledge of anosognosia mechanisms could have relevant clinical implications in patient care. Neuropsychological studies (Prigatano, 2009; Kaszniak and Edmons, 2010) support the hypothesis that anosognosia in MCI and AD reflects, at least in part, a failure to update the enduring self-awareness system based on the set of beliefs of one’s own capacities, attitudes, and traits in relation to those of others (Agnew and Morris, 1998; Morris and Hannesdottir, 2004), leading to a “petrification of the self” (Mograbi and Morris, 2013). Resting-state functional magnetic resonance imaging (rsfMRI) provides a way to investigate large-scale neuronal networks at the basis of how the brain is organized and functions. Among these, the *default mode network (DMN)* has emerged as a key network for self-awareness in diseased and healthy subjects: the involvement of cortical midline structures, including medial prefrontal cortex and medial posterior cingulate/precuneus, has been reported in studies requiring self-referential compared to other-referential judgments (Ries et al., 2007; Ruby et al., 2009; Mograbi and Morris, 2013; Zamboni et al., 2013b), and in functional and structural studies correlating the degree of awareness with structural (Rosen et al., 2010) or metabolic measures (Harwood et al., 2005; Vogel et al., 2005; Salmon et al., 2006). More recently, it has been demonstrated that anosognosia in amnestic MCI patients was associated with reduced connectivity between the precuneus and the bilateral inferior parietal lobes, left posterior cingulate cortex (PCC), and left orbitofrontal cortex, as well as between the right hippocampus and left middle temporal lobe and right fusiform cortex (Vannini et al., 2017). In a systematic review of articles using functional neuroimaging, authors concluded that the DMN of AD patients with anosognosia exhibits reduced within- and between-network connectivity, compared with AD patients without anosognosia and cognitively unimpaired participants; they also reported that during initial stages of cognitive decline in anosognosia, reduced neural activity is associated with the cortical midline regions, followed by the parietotemporal structures in later stages and culminating in frontotemporal dysfunction (Mondragon et al., 2019). In addition, a positive association between bilateral anterior cingulate cortex (ACC) and anosognosia was demonstrated in prodromal AD (Mondragon et al., 2021), meaning that the greater the anosognosia, the stronger the functional connectivity and suggesting that changes in ACC might precede changes found in DMN. Considering that the ACC is a central hub of the *salience network (SN)*, a resting-state network known to be involved in detecting, processing, and integrating internally and externally generated salient information (Seeley et al., 2007), it is plausible that an aberrant interaction between the DMN and the SN may characterize anosognosia in the AD continuum. A third network, often known as the *frontoparietal network* (FPN), has been implicated in the functional interplay between the DMN and the SN (Spreng et al., 2010), as well as in a range of executive functions, including working memory, performance monitoring, and planning (Niendam et al., 2012; Spreng and Schacter, 2012). It has been suggested that the disruption of the FPN may be associated with dysfunctions of the comparator system leading to anosognosia (Tagai et al., 2020). A reduced inter-communication between the DMN and the executive FPN was also demonstrated in a study exploring different forms of anosognosia in MCI and AD patients (Valera-Bermejo et al., 2021).

In the present study, we directly investigated the relationship between DMN, FPN, and SN with anosognosia in patients with AD and amnestic MCI, using independent component analysis applied to rsfMRI data. We hypothesized that anosognosia may be characterized by the disruption of the interplay between functional connectivity between large-scale cognitive networks.

## 2 Materials and methods

### 2.1 Participants

We included patients seen at the Oxford Memory Assessment Clinic of the John Radcliffe Hospital, Oxford, UK, between 2009 and 2011, who had received a diagnosis of amnestic MCI or probable AD, according to clinical criteria available at the time (Winblad et al., 2004; Mckhann et al., 2011). They also fulfilled subsequent revisions of core clinical criteria for MCI (Albert et al., 2011) or probable AD dementia (Mckhann et al., 2011). Participants underwent medical and neurological examination, neuropsychological assessment, and multimodal MRI scans. The degree of cognitive impairment was assessed by the Mini-Mental State Examination [MMSE, (Folstein et al., 1975) and the Montreal Cognitive Assessment MoCA, (Nasreddine et al., 2005)]. Participants’ study partners were interviewed to gain additional information about the participant’s cognitive, functional, and behavioral status. Prior, current, or past history of other neurological diseases, neurosurgery, or major psychiatric disorders (including depression), and the presence of behavioral disturbances other than anosognosia were considered exclusion criteria. Detailed demographic and neuropsychological characteristics are reported in Table 1. All participants and study partners signed informed consent prior participation. The study was conducted under ethical approval from the Central Office for NHS Research Ethics Committees and the Bristol Frenchay Research Ethics Committee (REC reference number 09/H0107/8).

**Table 1:** Demographic and neuropsychological characteristics of patients.

	Whole group (*n* = 30 MCI, 30 AD)	Aware (*n* = 30 MCI, 16 AD)	Unaware (*n* = 0 MCI, 14 AD)	Group comparison
Gender F:M	27:33	22:24	5:9	*p* = 0.4
Age (years)	75.4 (6.7; 57–87)	74.7 (± 7.23; 57–87)	77.5 (± 3.7; 67–83)	*p* = 0.07
Years of education	14 (3.5; 9–23)	13.7 (± 3.3; 9–23)	14.7 (± 4; 9–21)	*p* = 0.4
MMSE	24.5 (3.7; 14–29)	25.3 (± 3.2; 14–29)	22 (± 3.9; 15–28)	***p*** **= 0.02**
MoCA	19.5 (4.2; 6–27)	20.2 (± 4; 6–27)	18 (± 3.8; 10–26)	***p* = 0.05**
AQ-D	0.9 (15; −30 – 38)	−5.6 (± 10; −30 – 12)	22 (± 8.7; 16–38)	***p* < 0.001**

Mean values, standard deviation values and minimum - maximum range (in parenthesis) are reported. MMSE, mini mental state examination; MoCA, montreal cognitive assessment; AQ-D, anosognosia questionnaire – dementia. In bold, statistical significant results.

### 2.2 Measurement of anosognosia

The presence of anosognosia or impaired awareness was assessed by means of the discrepancy score evaluated by the Anosognosia Questionnaire Dementia (AQ-D) (Migliorelli et al., 1995), which consists of 30 questions divided into a cognitive and a behavioral section. The questionnaire consists of 30 questions divided into two sections: the cognitive part assesses cognitive function and performance in basic and instrumental activities of daily living, the behavioral part assesses changes in interests and mood. The same questions are administered to the patients (form A) and their caregivers (form B), who is blind to the patient’s answers, and the total AQ-D score is given by the difference between the patient and caregiver’s forms, so that the higher the score, the higher the degree of discrepancy, thus the anosognosia. Each question has a score ranging from 0 (never) to 3 (always); the minimum and maximum total scores obtainable on each form range from 0 to 90. The total AQ-D score is given by the difference between Form B and A. A positive final AQ-D score indicates that the caregiver has rated the patient as more impaired than the patient has rated himself or herself. Vice versa, a negative final AQ-D score indicates that the patient has overestimated his or her deficits. Higher scores indicate a reduced awareness of deficits, meaning that caregivers rated the patients as more impaired than did the patients. In previous studies using AQ-D form (Migliorelli et al., 1995; Amanzio et al., 2011, 2013), patients with a score at AQ-D of >32 were classified as being unaware, patients with a score of ≤14 were classified as being aware of their deficits, and patients who scored between 15 and 31 were classified borderline. In our study, as already used in previous papers from our group (Tondelli et al., 2022), we decided to classify patients with AQ-D < 14 as aware, and patients with AQ-D ≥ 14 as unaware, irrespective of their different degree of unawareness.

### 2.3 Demographic, neuropsychological and clinical characteristics analyses

Demographic, neuropsychological and clinical measures were compared between aware and unaware patients using descriptive statistics and group comparisons with parametric or non parametric statistical tests, depending on the distributional characteristics of the data.

### 2.4 Neuroimaging protocol

Scanning was performed the same day of the neuropsychological assessment at the University of Oxford OCMR Centre using a 3-T Trio Siemens MRI scanner equipped with a 12-channel head coil. Functional data were acquired using gradient-echo echo-planar (EPI) T2*-weighted images acquired along the transverse plane, parallel to the anterior to posterior commissural line (repetition time (TR) 2000 ms; echo time (TE) 28 ms; flip angle 89°; field of view (FOV) 192 mm; voxel size 3 × 3 × 3.5 mm; 180 volumes; acquisition time 6:04 min). Subjects were instructed to lie in dimmed light with their eyes open and not to think about anything.

A high-resolution T1-weighted 3D MP-RAGE anatomical image was also acquired for each patient to allow anatomical localization (TR = 2.040 ms; TE = 4.7 ms; flip angle 8°, FOV 192 mm; matrix size 256 × 192 × 124 voxels; voxel size 1 × 1 × 1 mm; acquisition time 5:56 min).

### 2.5 Resting-state fMRI data preprocessing and analyses

fMRI data analysis was performed using FSL tools (FMRIB Software Library, www.fmrib.ox.ac.uk/fsl). Resting state functional MRI data were analyzed using MELODIC (multivariate exploratory linear optimized decomposition into independent components, FSL http://www.fmrib.ox.ac.uk/fsl/melodic/). Functional volumes of each participant were preprocessed using motion correction, brain extraction, spatial smoothing (Gaussian kernel of FWHM 5 mm), and high-pass temporal filtering with a cutoff of 0.01 Hz. Functional volumes were registered to the individual’s structural T1 using boundary-based registration (Greve and Fischl, 2009) and then to standard space using non-linear registration (FNIRT). The FIX tool was used to denoise functional images from spurious signals (Salimi-Khorshidi et al., 2014). Preprocessed and denoised functional data were concatenated temporally and the resulting multi-subject dataset was fed into independent component analysis (ICA) and decomposed into 40 components. Biologically valid, non-artifactual resting state networks (RSNs) were identified by visual inspection (Yeo et al., 2011). The set of spatial maps from the group analysis was used to generate subject-specific versions of the spatial maps of the resting state networks, and associated timeseries, using dual regression (Nickerson et al., 2017).

#### 2.5.1 Resting-state network functional connectivity analyses

Based on our hypothesis, we selected the DMN, SN, and FPN as networks of interest (Yeo et al., 2011). We also included the occipital network (ON) as a control network about which we did not have an *a priori* hypothesis. For each of these 4 networks (DMN, SN, FPN, ON), a voxel-wise General Linear Models (GLM) was applied to the spatial maps using FSL’s randomise permutation-testing tool (5,000 permutations) in order to identify neural networks implicated in anosognosia (Nichols and Holmes, 2002). Given the lack of consensus on the most suitable method to determine anosognosia in dementia, we decided to apply two different methods: (i) correlation with the AQ-D scores, and (ii) group comparison between aware and unaware patients. Age, Mini-Mental State Examination (MMSE) scores, and grey matter volumes were included as regressor of no interest in all the GLMs (four correlation analyses, four group comparisons). Cluster-based thresholding was used to control for whole brain voxel-wise multiple comparisons, and a FWE-corrected significance threshold of *p* < 0.05 was applied to the suprathreshold clusters. Results were also explored at the *p* < 0.001, uncorrected statistical threshold.

##### 2.5.1.1 Inter-network functional connectivity analyses

Individual ICA time-courses associated with DMN, SN, and FPN networks were considered. A low-pass filter with a passband frequency of 0.15 Hz was applied to the time-courses using the MATLAB (The MathWorks Inc., Natick, Mass) “lowpass” function (Josephs and Henson, 1999; Tong et al., 2019). Considering the importance of DMN as a key network for self-awareness in diseased (Nichols and Holmes, 2002; Ries et al., 2007; Zamboni et al., 2010, 2013a) and healthy subjects (Davey et al., 2016), partial correlation was used to assess inter-network functional connectivity between DMN and SN, and between DMN and FPN, separately for aware and unaware patients (Smith et al., 2011). Then, the resulting inter-networks functional connectivity values were correlated to each other to assess whether the functional connectivity of DMN with SN was significantly associated with the connectivity strength between DMN and FPN.

## 3 Results

**Figure 1:**
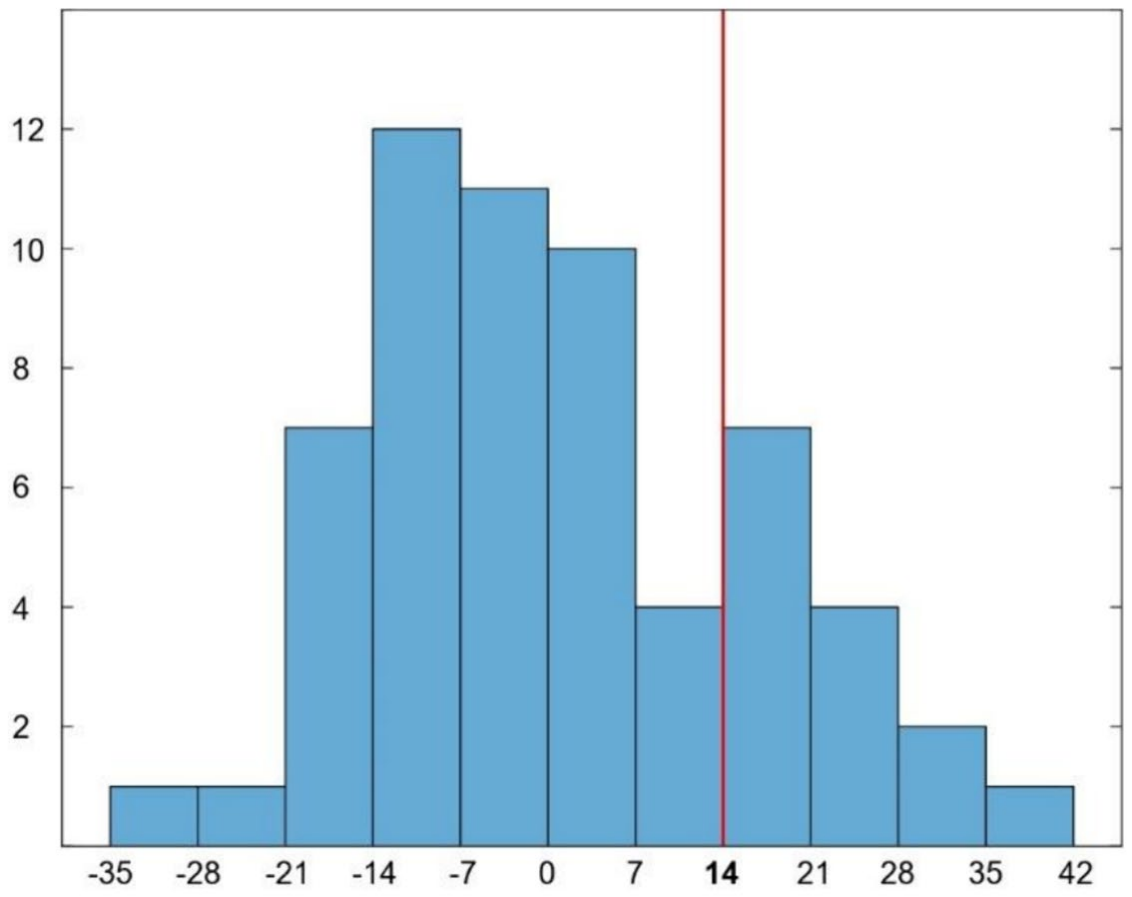
Histogram representing the distribution of AQ-D scores in our patients. Red line indicates the cut-off (score ≥ 14) for disease awareness.

Sixty patients were recruited (27 females; mean age 75.4 ± 6.7 years; mean education 14 ± 3.5 years of school attendance). The mean AQ-D score was 0.9 ± 15 (Figure 1). According to the established cutoff, 14 patients had anosognosia (unaware), whereas 46 patients had preserved awareness of their cognitive deficits (aware). There were no significant differences in age, years of education or gender between aware and unaware patients. Mean scores of cognitive impairment were 25.3 ± 3.2 for aware and 22 ± 3.9 for unaware patients for the MMSE, and 20.2 ± 4 for aware and 18 ± 3.8 for unaware patients for the Montreal Cognitive Assessment (MoCA).

According to previous studies suggesting the presence of hypernosognosia in amyloid-positive healthy subjects (Vannini et al., 2017), we explored whether high level of awareness were related to global cognitive impairment (evaluated by MMSE). We found that hypernosognosic patients (AQ-D discrepancy score < −14; *N* = 9, mean MMSE = 25.9) show lower cognitive impairment (*p* = 0.03) in comparison to unaware patients (AQ-D ≥ 14; *N* = 14, mean MMSE = 22), but not statistically significant different cognitive impairment (*p* = 0.8) compared to aware patients (AQ-D -14 < x < 14; N = 37, mean MMSE = 25).

### 3.1 Anosognosia and resting-state network functional connectivity

The DMN, SN, FPN and ON were identified and reported in Supplementary Figure S1.

#### 3.1.1 Default mode network

**Figure 2:**
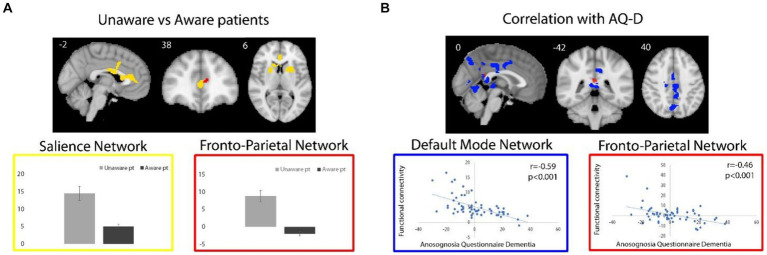
**(A)** For unaware relative to aware patients, regions of significant higher functional connectivity with the SN and FPN are shown in yellow and red, respectively. Box plots represent average (and standard deviations) of the parameter estimates values (y axes) extracted from brain regions with significant group differences. **(B)** Regions of negative correlation between AQ-D scores and functional connectivity of DMN and FPN are shown in blue and red, respectively. Functional connectivity measures were extracted from the resulting regions and plotted for visualization purposes. Spatial maps are superimposed on the MNI152 standard space template image.

Across all patients, functional connectivity of the DMN negatively correlated with the AQ-D score in the bilateral middle cingulate cortex (MCC; 1,447 voxels; T-max 4.86; MNI coordinates, x, y, z = 10, −22, 32), posterior cingulate cortex/precuneus (PCC/PCU; 700 voxels; T-max 4.4; x, y, z = 16, −64, 24), cerebellum, right inferior occipital gyrus and left thalamus (699 voxels; T-max 4.45; x, y, z = −2, −28, −2; Figure 2B). In other words, the association between these regions and DMN is lower in patients with higher anosognosia.

The direct comparison of DMN connectivity between unaware versus aware patients revealed decreased functional connectivity in the MCC in unaware patients (50 voxels; T-max 4.49; x, y, *z* = 14, −22, 32), when exploring results at a lower, but still reasonable statistical threshold (*p* < 0.001), uncorrected for multiple comparisons. No significant clusters survived for from the unaware > aware contrast.

#### 3.1.2 Salience network

Across all patients, functional connectivity of the SN positively correlated with the AD-Q score at an uncorrected statistical threshold of p < 0.001 in the right superior (382 voxels; T-max 4.11; MNI coordinates, x, y, z = 24, 22, 60) and medial superior (208 voxels; T-max 4.18; x, y, z = 18, 60, 34) frontal gyri, right orbitofrontal cortex and anterior insula (51 voxels; T-max 3.3; x, y, z = 30, 24, −18).

Functional connectivity of the SN was increased in unaware relative to aware patients in the anterior cingulate cortex (ACC), dorsal-ACC, and basal ganglia (right caudate head and bilateral putamen) (1,256 voxels; T-max 4.86; x, y, z = −4, 10, 36; Figure 2A). In other words, patients with anosognosia had increased connectivity of these regions with the SN. The aware > unaware contrast did not yield any significant cluster.

#### 3.1.3 Frontoparietal network

As for the FPN, no cluster survived the selected statistical threshold neither for correlation analysis nor for the comparison between unaware and aware patients. Nevertheless, at the uncorrected statistical threshold of p < 0.001, the functional connectivity of the FPN negatively correlated with the AQ-D score in the PCC (16 voxels; T-max 3.47; x, y, z = 0, −42, 20; Figure 2B), i.e., an area in which there was also a significant negative correlation with the DMN, as well as in the right orbitofrontal cortex (24 voxels; T-max 3.24; x, y, z = 16, 32, −20). Also, at the uncorrected statistical threshold of p < 0.001, the functional connectivity of the FPN was stronger in unaware relative to aware patients in the ACC (8 voxels; T-max 3.6; x, y, z = −10, 42, 12, Figure 2A), i.e., an area in which there was also significantly increased functional connectivity of the SN. No significant clusters were observed in from the aware > unaware contrast.

#### 3.1.4 Occipital network

No regions survived the selected corrected and uncorrected statistical thresholds in correlation analysis or comparisons between unaware and aware patients.

### 3.2 Anosognosia and inter-network functional connectivity

For each partial correlation, the mean Pearson’s *r*, the standard deviation, and minimum–maximum values were reported.

The average partial correlation between DMN and SN was −0.077 ± 0.273 (−0.714–0.498) for aware patients, and 0.022 ± 0.243 (−0.408–0.351) for unaware ones. The average partial correlation between DMN and FPN was −0.052 ± 0.260 (−0.708–0.364) for aware patients, and − 0.020 ± 0.207 (−0.300–0.452) for unaware ones. SN and FPN showed an average partial correlation of −0.070 ± 0.293 (−0.845–0.489) for aware patients, and of −0.062 ± 0.293 (−0.589–0.429) for unaware ones.

**Figure 3:**
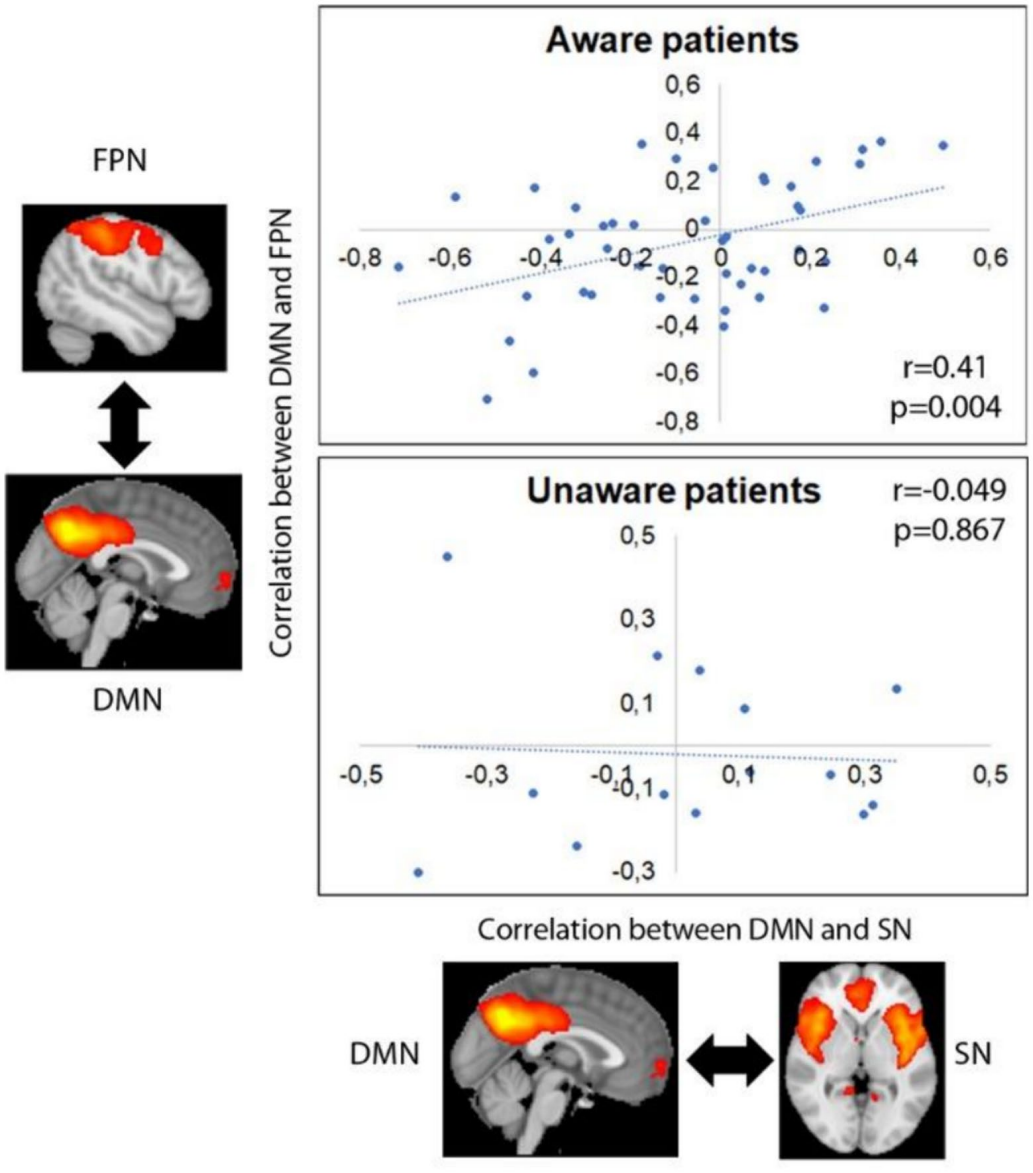
Correlation between internetwork functional connectivity in aware and unaware patients. Images represent average group maps of both aware and unaware patients obtained using independent component analysis.

Functional connectivity between DMN and SN was positively correlated with functional connectivity between DMN and FPN in aware patients (*r* = 0.421, *p* = 0.004), but not in unaware ones (*r* = −0.049, *p* = 0.867) (see Figure 3).

## 4 Discussion

In our study we investigated large-scale functional brain networks connectivity associated with anosognosia in AD. We found that anosognosia was associated with (i) DMN-related low functional connectivity, particularly in posterior medial cortical regions including the PCC and MCC, (ii) high SN-related functional connectivity in anterior medial cortical regions including the ACC as well the basal ganglia, and (iii) opposite effects regarding functional connectivity of the FPN, i.e., low connectivity with posterior medial cortical regions (PCC) and high connectivity with anterior medial cortical regions (ACC). Moreover, our results showed that the strength of functional connectivity between DMN and SN was significantly correlated with the connectivity strength between DMN and FPN in aware patients only, supporting the hypothesis that anosognosia may be associated with a disruption of the large-scale connectivity patterns between these networks.

In line with previous literature (Zamboni et al., 2013b; De Bruycker et al., 2017; Vannini et al., 2017; Valera-Bermejo et al., 2021), we observed a significant negative correlation between functional connectivity within DMN nodes and awareness, meaning that higher levels of anosognosia were associated with lower functional connectivity of these areas. In particular, we found decreased connectivity with PCC and PCU, key structures of the DMN. Association between PCC/PCU and awareness processes are consistent in literature: task-based fMRI studies conducted in healthy subjects have constantly demonstrated the activation of PCC in relation to self-appraisal (Johnson et al., 2002) and autobiographical memory (Fink et al., 1996; D’argembeau et al., 2005). Several studies demonstrated a decreased functional connectivity between the PCC and the hippocampus in anosognosic patients (Perrotin et al., 2015; Vannini et al., 2017); in addition, PCC was also found to be implicated in implicit awareness mechanisms when using a modified Stroop emotional task (Tondelli et al., 2022). Cavanna and Trimble (2006) hypothesized that self-appraisal processes were driven by PCC activity involved in retrieval and integration of mental representations, therefore, if applied to Cognitive Awareness Model (CAM) (Agnew and Morris, 1998; Lenzoni et al., 2020), PCC may play a role in integration and comparison of current information with stored autobiographical information (Salmon et al., 2024). Decreased functional connectivity was also found in MCC with the DMN component when considering both AQ-D correlation analysis and comparison between aware and unaware patients. Major projections of MCC are known to be widespread over all cingulate cortex and parietal regions (Vogt, 2016), suggesting intense connection of this region with hub nodes of human cognition. In addition, MCC has been proposed as a key region for social cognition and social behavior playing an important role in predicting and monitoring the outcomes of one’s own and others’ decisions (Apps et al., 2013).

Interestingly, involvement of medial and lateral temporal regions was not revealed in our study. Several neuroimaging studies showed that middle temporal gyrus is implicated in autobiographical memory suggesting that this area could be a key region for the personal database in the CAM leading to “the petrification of the self” as a core feature of anosognosia in AD (Salmon et al., 2024). Previous fMRI studies investigating network connectivity in anosognosia demonstrated temporal cortical connectivity involvement when using measures of memory anosognosia: Antoine et al. reported disconnection within the medial temporal subsystem of the default mode network, subserving episodic memory processes, using a Memory Awareness Rating Scale (Antoine et al., 2019); Valera-Bermejo et al. showed that memory anosognosia scores were associated with selective lower frontotemporal connectivity and higher parietotemporal connectivity, whereas total anosognosia scores were associated with large-scale network alterations, namely reduced left-FPN expression in the left posterior cingulate, reduced right-FPN expression in the left inferior lingual gyrus and adjacent inferior occipital cortex, and increased right-FPN expression in the right anterior cingulate (Valera-Bermejo et al., 2021). Therefore, activation/deactivation of subregions of the DMN may be differentially elicited for mnemonic, not mnemonic, and global anosognosia. More precisely, our results suggest that the posterior DMN may be implicated in higher-level, comparative mechanisms common to mnemonic and global anosognosia. It is plausible that the other subregions of the DMN and the temporal lobes may be, instead, involved in lower-level mechanisms of mnemonic anosognosia more related to semantic and episodic memory.

When exploring the SN, significant increased functional connectivity was observed in the ACC, dorsal-ACC, and basal ganglia in unaware relative to aware patients. ACC is a major hub of the SN with a key role in processing and integrating internal and external inputs for decision-making (Seeley et al., 2007). As suggested by Salmon and co-authors (Salmon et al., 2024), ACC activity might be involved in higher-order processes for self-referencing (related to the metacognitive awareness system in the CAM model). Our results suggest that the greater the anosognosia, the stronger the SN-related ACC functional connectivity. Mondragon and co-authors had also found that prodromal AD patients exhibit a positive association between anosognosia and brain connectivity in the bilateral ACC (Mondragon et al., 2021). Similarly, Valera-Bernejo and coauthors showed, with a seed-based functional connectivity analysis approach, that higher anosognosia was associated in MCI and AD patients with stronger functional connectivity pattern between anterior cingulate and right dorsolateral prefrontal cortex (Valera-Bermejo et al., 2021). The same authors, using a large-scale networks connectivity methodology, found positive associations between total anosognosia scores and an anterior division of DMN in the right anterior cingulate, suggesting that total anosognosia might be expression of abnormally increased up-regulation of DMN-salience inter-network connectivity in relation to reduced self-processing of internal stimuli in comparison to external ones (Valera-Bermejo et al., 2021). Our findings are in line with the hypothesis that anosognosia may be the result of a “network’s imbalance” between large-scale cognitive networks: a decreased DMN-related functional connectivity, associated with an “adaptive (or maladaptive)” increased SN-related functional connectivity. We also found a higher SN-related functional connectivity in the basal ganglia of patients with anosognosia. In a study by Shany-Ur (Shany-Ur et al., 2014), authors found that overestimation of functioning was related to several subcortical structures including the putamen, thalamus, caudate, pons, and midbrain. The functional interplay between cortical midline structures and subcortical structures may also be involved in transforming lower level, interoceptive bodily sensations, and representations of self into higher-level self-referential mental representations (Northoff et al., 2006). When considering a more tolerant statistical threshold, a positive correlation between the AQ-D score and the SN-related connectivity of medial superior frontal gyrus, right orbitofrontal cortex (OFC)/anterior insula (AI) and middle/superior frontal gyrus was observed. Among these regions, the OFC is known to be a key brain area in emotion processing and the representation of reward value (Rolls, 2016). Interestingly, increased functional connectivity of the lateral OFC with brain areas that include the PCU, PCC, and angular gyrus was found in patients with depression and was found to be reduced in medicated patients (Rolls, 2016). Similarly, also in our study, higher level of anosognosia was found to be associated with higher connectivity of SN with OFC, confirming the role of OFC in self-evaluation. AI is well known as a major node of SN: the interplay between ACC and frontoinsular cortex is involved in detecting, integrating, and filtering relevant interoceptive, autonomic, and emotional information, and has thus been proposed as a key structure for awareness processes.

Finally, although less statistically significant, the investigation of FPN-related functional connectivity showed a negative correlation with the AQ-D score in the PCC and increased functional connectivity in the ACC in unaware relative to aware patients. The association of anosognosia with divergent patterns of posterior/decreased versus anterior/increased FPN-related functional connectivity overlaps with the association of anosognosia with the DMN and SN separately in the same medial cortical regions, i.e., lower DMN-related functional connectivity in the PCC and higher SN-related functional connectivity in the ACC. This suggests that the FPN activity may variably couple with DMN and SN according to the degree of awareness/anosognosia. This is in line with the proposed role of the FPN in facilitating the functional interplay between the DMN and SN in supporting goal-directed cognition (Spreng et al., 2013). Note that the FPN overlaps largely with, and is often considered a synonym of, the so-called Central Executive Network (CEN) (Menon, 2011), which has been demonstrated to be crucial for actively maintaining and manipulating information in working memory, for rule-based problem solving and for decision making in the context of goal-directed behavior. According to a recently proposed taxonomy of macro-scale functional resting networks (Uddin et al., 2019), which introduces the concept of dorsal FPN as an attention network and of lateral FPN as a control network, the FPN connectivity map that emerged from our data overlaps with the dorsal, attentional FPN. We speculate that different subsystems of this network might variably couple with the DMN and SN nodes depending on whether the self-process demand is more related to internal (DMN) or external (SN) experiences. In other words, the FPN may play the role of a cortical mediator between the other two networks, in support of cognitive and meta-cognitive processes.

### 4.1 A speculative link to the cognitive awareness model

**Figure 4:**
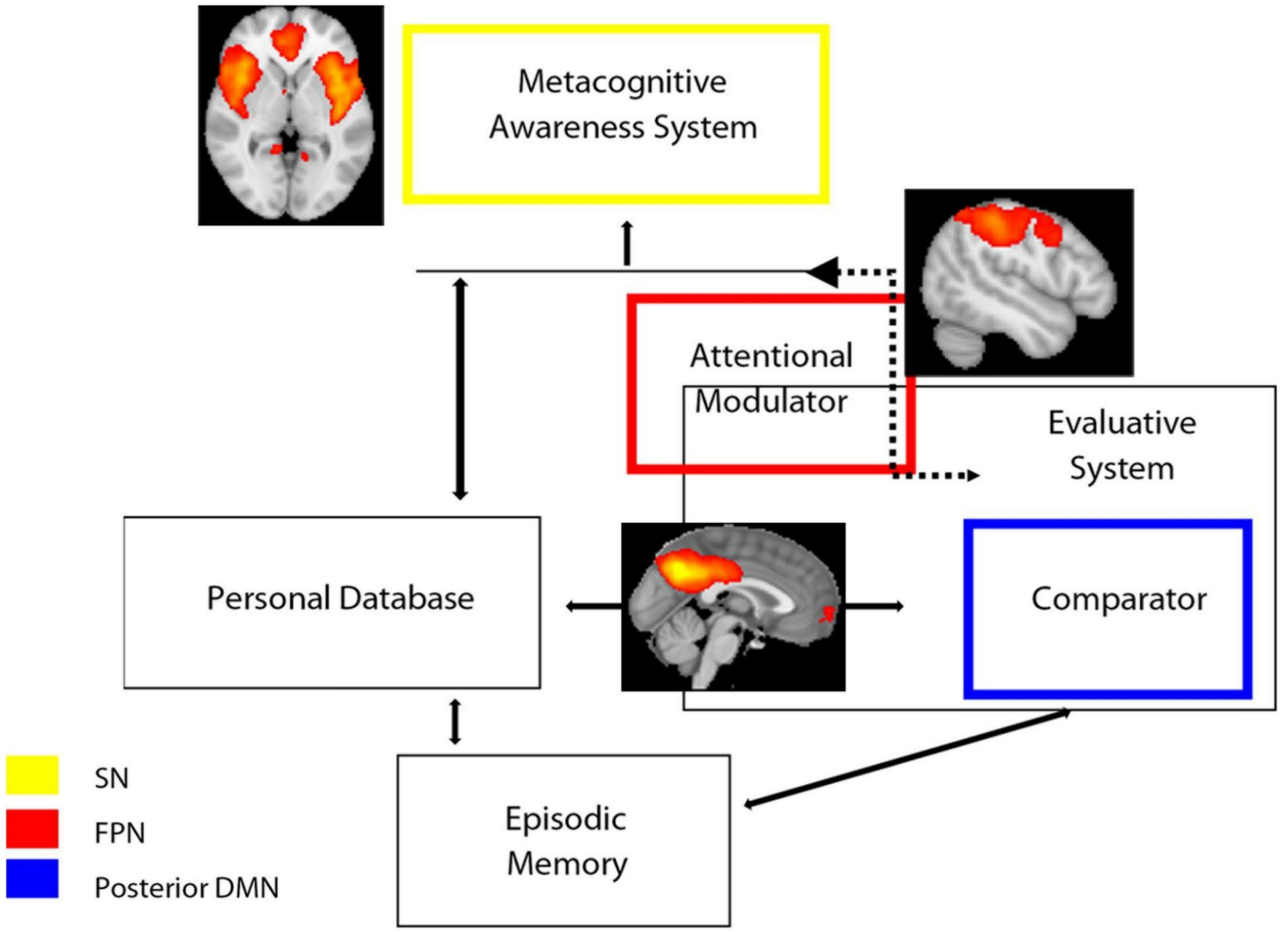
Schematic representation of the hypothesized model of awareness based on CAM model (Antoine et al., 2019; Lenzoni et al., 2020) superimposed with functional connectivity large-scale networks and the results of the present study. SN, salience network; FPN, frontoparietal Network; DMN, default mode network.

The Cognitive Awareness Model (CAM) has been described as a model of awareness of cognitive functioning (Agnew and Morris, 1998; Morris and Mograbi, 2013; Uddin et al., 2019; Lenzoni et al., 2020). The key notion of this model is that the knowledge about one’s own performance is monitored by a Comparator System (CS), which contrasts the incoming information with that stored in a Personal Database (PDB). If there is a mismatch between the two sources of data, the resulting information is released via a further mechanism, namely the Metacognitive Awareness System (MAS), to provide consciousness on one’s own behavior. Therefore, one would expect that self-awareness (and its counterpart anosognosia) rely upon functionally correlated brain systems that support communication between an “external” source of information and “internal” personal knowledge. In the light of our results, we speculatively adapted our results on large-scale networks dynamics to the CAM model (Figure 4). In this adaptation, the posterior component of the DMN, particularly the PCC, is part of the comparator system, which also includes other regions such as the basal ganglia. The SN, particularly the ACC, is instead part of the MAS, which provides consciousness and higher metacognitive awareness. Finally, the FPN functions as an attentional modulator, which flexibly couples with the DMN or SN networks depending on whether the task demand is internal or external, respectively. Imbalance of these networks’ connectivity translates into a disruption of awareness mechanisms: decreased DMN-related functional connectivity (involved in inner experience and self-reflection) is associated to excessively increased SN-related functional connectivity (involved in mechanisms of integration of external salient data). We speculate that, in anosognosia, an imbalance/disruption between DMN and SN occurs: if DMN fails to produce and sustain the updating and evaluation of new information in relation to personal knowledge (because of or in association with a failure of DMN temporal nodes for example, corresponding to personal database and episodic memory systems), the FPN might modulate/shift attention to external salient data, producing hyperconnectivity (probably mal-adaptive rather than compensatory) in SN nodes.

## 5 Limitations

We recognize some limitations for our study. First, we used only a single method for anosognosia assessment, and it is well known that different measures may lead to explore different aspects of awareness (Tondelli et al., 2018). Awareness is not an all-or-nothing phenomenon; it may be that patients are aware of some type of impairment but not of others. Whereas measurements based on examiner’s ratings usually refer to global and general unawareness (including cognitive, behavioral, and functional abilities), measurement of discrepancy and performance variably refer to different specific domains depending on the questionnaire and neuropsychological tests adopted. All these approaches have significant limitations and there is no consensus on the most suitable method to determine anosognosia in dementia; nevertheless, the discrepancy score is currently the most accepted methodological approach (De Ruijter et al., 2020). Another limitation of our study is that we included patients with a clinical diagnosis of probable AD and amnestic MCI patients according to clinical criteria available at the time of recruitment and not supported by cerebrospinal fluid or PET-based biomarkers. All patients did however have hippocampal atrophy (Zamboni et al., 2013a) and subsequent clinical follow-up confirmed progression of all MCI to probable AD dementia. To partially address the issue of clinical heterogeneity and the fact that the severity of cognitive impairment is differently distributed across the aware and unaware groups, all the functional connectivity analyses were performed including a measure of disease severity (the MMSE) and age as covariates of no interest with the aim of removing their confounding effect on imaging data. Moreover, grey matter maps were also added as additional voxel-wise covariate in the imaging analysis in order to remove the influence of atrophy, which is known to parallel the severity of cognitive impairment, on functional connectivity measures.

## 6 Conclusion

In the present study, investigating the relationship between anosognosia and large-scale brain networks in patients with AD, we propose that anosognosia may result from an “imbalance” between abnormally decreased functional intrinsic connectivity in the DMN paired with abnormally increased functional intrinsic connectivity in the SN. This imbalance might be facilitated by the activity of frontoparietal circuits that may variably couple with the default and salience networks according to task demand. Dysfunctional network dynamics may lead to anosognosia irrespective of neuropathology, and thus should be considered markers of a symptom rather than markers of a specific disease. Future studies including patients with other neurocognitive disease (for example frontoparietal dementia) should be performed to address and further investigate the generalizability of our results.

## Data availability statement

The raw data supporting the conclusions of this article will be made available by the authors, without undue reservation.

## Ethics statement

The studies involving humans were approved by Central Office for NHS Research Ethics Committees and the Bristol Frenchay Research Ethics Committee (REC reference number 09/H0107/8). The studies were conducted in accordance with the local legislation and institutional requirements. The participants provided their written informed consent to participate in this study.

## Author contributions

MT: Conceptualization, Data curation, Formal analysis, Funding acquisition, Investigation, Methodology, Project administration, Resources, Software, Supervision, Validation, Visualization, Writing – original draft, Writing – review & editing. DB: Conceptualization, Data curation, Formal analysis, Funding acquisition, Investigation, Methodology, Project administration, Resources, Software, Supervision, Validation, Visualization, Writing – original draft, Writing – review & editing. RM: Formal analysis, Methodology, Writing – review & editing. CC: Writing – review & editing. CG: Writing – review & editing. CM: Writing – review & editing. GP: Formal analysis, Methodology, Writing – review & editing. AC: Data curation, Writing – review & editing. GZ: Conceptualization, Data curation, Formal analysis, Funding acquisition, Investigation, Methodology, Project administration, Resources, Software, Supervision, Validation, Visualization, Writing – original draft, Writing – review & editing.
