# Supplementary material for: Resting-state networks and anosognosia in Alzheimer’s disease
Source: Front Aging Neurosci. 2024 Jun 5;16:1415994. doi: 10.3389/fnagi.2024.1415994 (PMC11188402; doi:10.3389/fnagi.2024.1415994)
Supplement: Supplementary file 1 [file Data_Sheet_1.docx]

**Supplementary Material**

**Supplementary Figure 1 caption**: The average group maps of the whole group are displayed superimposed on the MNI152 standard space template image obtained using a data-driven approach. R refers to the right, L to the left hemisphere, S to superior, and I to inferior.
