# Supplementary figures and images for: Resting-state networks and anosognosia in Alzheimer’s disease
Source: Front Aging Neurosci. 2024 Jun 5;16:1415994. doi: 10.3389/fnagi.2024.1415994 (PMC11188402; doi:10.3389/fnagi.2024.1415994)

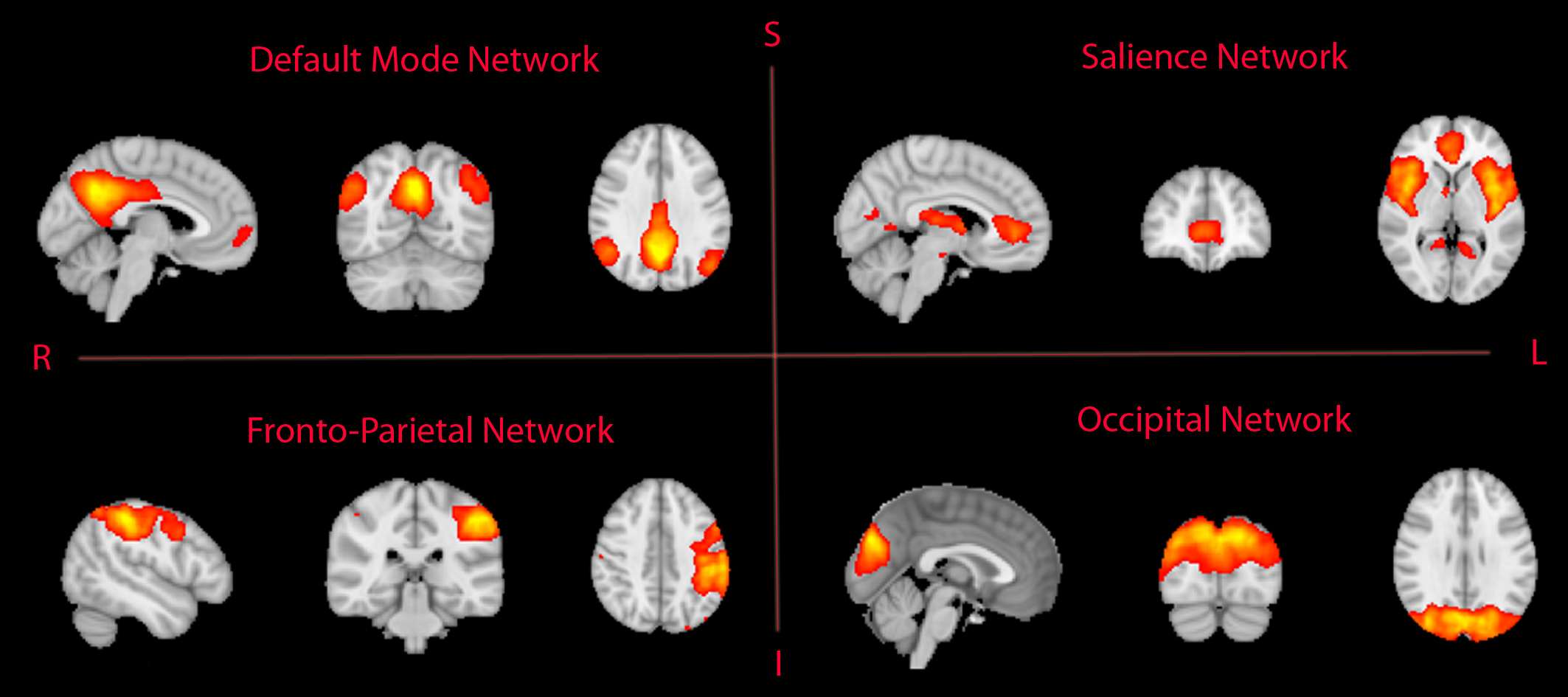

Supplement: Supplementary file 2 [file Image_1.JPEG]
